# Understanding the Role of Clinical Decision Support Systems Among Hospital Nurses Using the FITT (Fit Between Individuals, Tasks, and Technology) Framework: Qualitative Study

**DOI:** 10.2196/76025

**Published:** 2025-09-25

**Authors:** Matthijs Berkhout, Koen Smit, Danielle Sent, Rob Kusters, Johan Versendaal, Thijs van Houwelingen

**Affiliations:** 1 Research Group on Digital Ethics Research Centre for Learning and Innovation University of Applied Sciences Utrecht Utrecht The Netherlands; 2 Faculty of Science Open University of the Netherlands Heerlen The Netherlands; 3 Research Group on Artificial Intelligence University of Applied Sciences Utrecht Utrecht The Netherlands; 4 Jheronimus Academy of Data Science Eindhoven University of Technology and Tilburg University ’s-Hertogenbosch The Netherlands; 5 Research Group on Technology for Healthcare Innovations Research Centre for Healthy and Sustainable Living University of Applied Sciences Utrecht Utrecht The Netherlands

**Keywords:** clinical decision support systems, CDSS, nursing, technology adoption, qualitative research, FITT framework, usability, hospital nursing

## Abstract

**Background:**

Clinical decision support systems (CDSSs) have gained prominence in health care, aiding professionals in decision-making and improving patient outcomes. While physicians often use CDSSs for diagnosis and treatment optimization, nurses rely on these systems for tasks such as patient monitoring, prioritization, and care planning. In nursing practice, CDSSs can assist with timely detection of clinical deterioration, support infection control, and streamline care documentation. Despite their potential, the adoption and use of CDSSs by nurses face diverse challenges. Barriers such as alarm fatigue, limited usability, lack of integration with workflows, and insufficient training continue to undermine effective implementation. In contrast to the relatively extensive body of research on CDSS use by physicians, studies focusing on nurses remain limited, leaving a gap in understanding the unique facilitators and barriers they encounter.

**Objective:**

This study aimed to explore the facilitators and barriers influencing the adoption and use of CDSSs by nurses in hospitals, using an extended Fit Between Individuals, Tasks, and Technology (FITT) framework.

**Methods:**

A qualitative study was conducted using semistructured interviews with 22 nurses from across the Netherlands, representing 3 hospital types: general (n=9), top-clinical (n=12), and academic (n=1). The sample included a diverse mix of practicing nurses, nurses-in-training, and clinical nurse information officers, with clinical experience ranging from 1.5 to 38 years. Interview transcripts were analyzed thematically, beginning with an inductive coding approach to identify key factors. These were then categorized deductively using the extended FITT framework. In total, 988 code instances were examined. To ensure analytical rigor, the coding process was separately conducted by 2 researchers and reviewed by an expert panel.

**Results:**

A total of 26 distinct factors were identified, categorized into 4 FITT dimensions: technology-individual, technology-task, task-individual, and organizational context. Of these, 11 factors were facilitators (eg, cognition, clarification, and prevention), 7 were barriers (eg, alarm fatigue, poor design, and limited digital proficiency), and 8 were both facilitators and barriers depending on the context (eg, acceptance, workload, and training). In addition, key value tensions emerged, such as the balance between standardization and professional autonomy, and the trade-off between enhanced decision support and increased administrative burden.

**Conclusions:**

The findings underscore the complexity of CDSS adoption in nursing practice, highlighting the interaction of facilitators and barriers across FITT dimensions. Practical recommendations include participatory design processes, targeted training programs, advanced alert management systems, and strong organizational support. Addressing value tensions and aligning CDSS functionality with nurses’ workflows can enhance adoption and optimize patient outcomes.

## Introduction

Information technology (IT) has become increasingly important in all industries, including health care. In the past 25 years, a wide variety of information systems were invented and implemented to aid health care professionals in decision-making, including clinical decision support systems (CDSSs). CDSSs are intended to improve health care delivery by enhancing medical decisions with targeted clinical knowledge, patient information, and other health information [1,2]. Examples of CDSSs used in health care are diagnosing health problems and medication-prescribing [3,4]. One of the use cases for using a CDSS is the prevention of medical errors [2-4]. Overall, the role of CDSSs in the health care sector has received substantial recognition, significantly impacting patient care quality, safety, and efficiency [2,5]. One critical user group of these systems is nurses, whose central role in direct patient care, monitoring, and coordination between health care teams makes CDSS integration essential for improving workflow efficiency and patient outcomes.

The use of CDSSs diverges significantly among various health care professionals in hospitals, such as physicians and nurses, according to their distinct roles and responsibilities. Physicians predominantly use CDSSs to aid diagnostic reasoning and to optimize treatment plans [2,6]. In this context, physicians rely on these systems to filter vast amounts of clinical data to gather precise diagnoses, suggest potential treatment options, and predict patient responses to different treatments. On the other hand, nurses employ CDSSs for a slightly different set of tasks. As emphasized in a study by Ruland and Bakken [7], nurses face significant challenges in managing complex care tasks, including continuous patient monitoring, timely detection of clinical deterioration, and prioritizing care under time pressure. CDSSs can help address these challenges by offering timely, structured support in areas such as care planning, infection control, and wound management. A widely used application of CDSSs is the filtering of alarms in intensive care for neonatal intensive care units, where the system helps distinguish clinically relevant alerts, such as those related to infusion pumps or critical vital signs, from nonurgent ones caused by, for instance, loose cables.

Despite these demonstrated benefits, the literature on CDSS adoption by nurses remains relatively limited and fragmented. A recent rapid review synthesizing 21 studies concluded that while CDSSs improve nursing decision-making, workflow efficiency, and patient outcomes, they also present persistent barriers such as alert fatigue, workflow misalignment, and limited adherence to system recommendations [4,8,9]. While existing research provides valuable insights into specific functionalities and adoption barriers, there is still a lack of systematic understanding of how CDSSs fit into the day-to-day clinical realities of nurses. Recent qualitative and mixed methods reviews call for more in-depth exploration of nurses’ experiences and perspectives, as these are often underrepresented compared to physician-centric studies [8].

**Figure 1:**
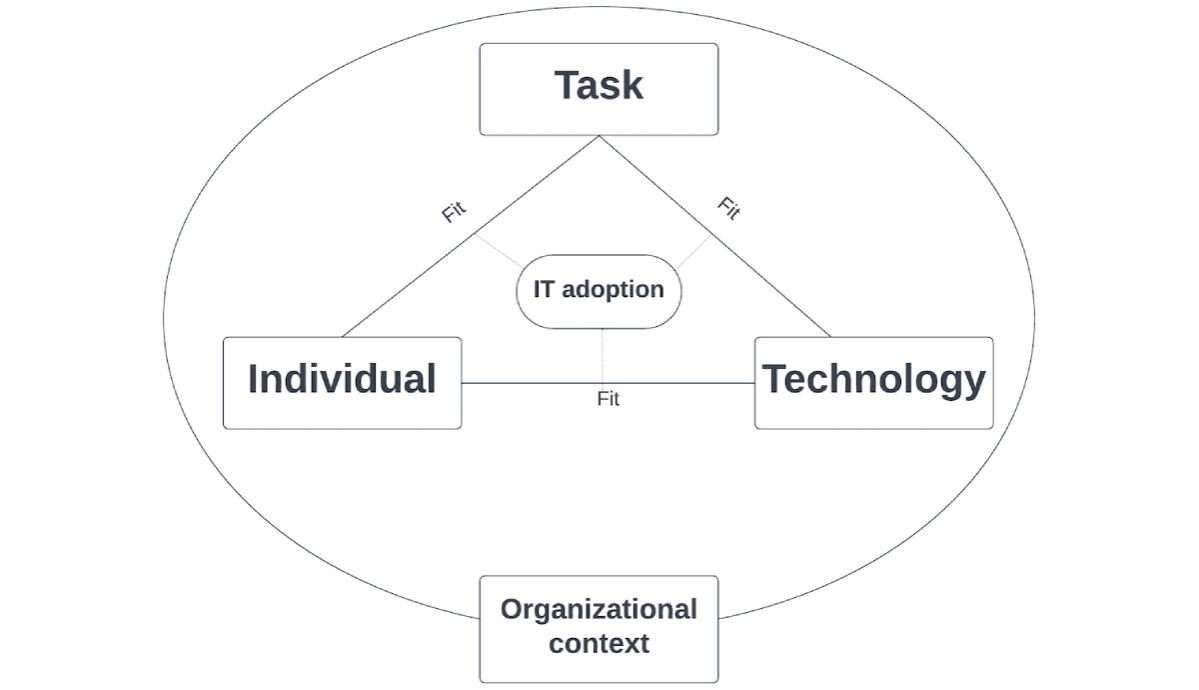
Extended FITT (Fit Between Individuals, Tasks, and Technology) framework [10,11,15]. IT: information technology.

To better capture this complexity, we use the extended Fit Between Individuals, Tasks, and Technology (FITT) framework, which states that the success of health IT adoption depends on the alignment between user characteristics, task demands, technology features, and organizational context (Figure 1) [10,11]. The FITT model has been applied and validated in various health IT studies, including electronic health record (EHR) and telemonitoring adoption, and is particularly well suited to understanding technology adoption in complex, team-based clinical environments [10,12-14]. Compared to behavioral models such as the Unified Theory of Acceptance and Use of Technology (UTAUT), FITT places greater emphasis on the practical interaction between people, systems, and work processes, making it especially relevant for exploring the nursing context [10,15]. The disconnect between the theoretical advantages of CDSSs and their real-world application in nursing highlights the urgent need to address these challenges [16].

Prior reviews cover clinicians broadly or cover nonhospital settings, with limited qualitative evidence from hospital nurses and little integration of findings within an explicit implementation lens [8,17]. Framework and feature scoping reviews inform *what* may help adoption but not *how* ward-level nursing work experiences map onto domains such as FITT [18,19]. We contribute nurse-focused qualitative evidence, map facilitators, barriers, and value tensions onto FITT, and compare patterns across Dutch hospital types.

This paper aims to address the gaps in our understanding of the potential complex and relatively unexplored dynamics associated with decision support systems usage in a nursing context. Through the extended FITT framework, we explore how individual nurse factors, executing the task of decision-making, and the supporting technology interact to facilitate or hinder effective decision support system usage.

Therefore, the research question addressed in this paper is “What are the facilitators and barriers for hospital nurses regarding the use of a clinical decision support system?”

From a theoretical perspective, the goal is to contribute to the body of knowledge that informs health care policy and system design, fostering environments where technology effectively supports and enhances patient care delivery in hospitals. For example, studies have shown that user-centered design and tailored technology adaptation enhance the usability and effectiveness of CDSSs [6]. By using the extended FITT framework, we can identify how CDSSs can be better tailored to fit the specific needs of users, such as nurses and physicians, reducing barriers and enhancing facilitators for more seamless integration into clinical workflows [2]. This approach ensures that technology aligns with real-world practices, leading to improved patient outcomes [10].

From a practical perspective, this study aims to contribute to the limited body of research on the implementation and use of CDSSs in a hospital nursing context. By offering insights into practical strategies to overcome barriers and leverage facilitators, this research provides valuable guidance for (quality) nurses, health care IT developers, and management. A better understanding of CDSS design and adoption specifically tailored to nurses’ needs can support better integrated workflows and enhance both nursing practice and patient outcomes.

## Methods

### Design

In this qualitative study, semistructured interviewing was chosen as the primary data collection method to operationalize the FITT framework because it allows for in-depth exploration of nurses’ experiences, perceptions, and challenges with CDSSs in a hospital setting. Given the complex nature of the interactions between individuals, tasks, and technology, interviews offer a nuanced understanding that is essential for capturing the subjective experiences that other methods, such as secondary sources, may not provide.

The study specifically focuses on a CDSS in a hospital setting. Our research methodology was designed with an orientation toward the extended FITT framework, which suggests that successful technology implementation and use is largely determined by the fit between 3 aspects and the organizational context [10,11]. This framework was particularly relevant to our goal of identifying facilitators and barriers in the use of CDSSs by nurses. By focusing on the alignment between the individual users (nurses), their tasks, the technology itself, and the organizational context, we are able to explore how CDSSs either support or hinder their work. This approach enabled us to find specific factors that act as facilitators, enhancing system usability and integration, as well as barriers that impede effective use.

### Setting

The interviews were conducted face-to-face at the participants’ own hospital in the Netherlands. In the Dutch health care system, hospitals are generally categorized into 3 types: general hospitals, top-clinical hospitals, and academic hospitals. General hospitals provide a broad range of standard medical services and are typically smaller in scale. Top-clinical hospitals offer more specialized care and often serve as regional centers for complex procedures; they are also typically affiliated with teaching programs. Academic hospitals are university-affiliated institutions that combine advanced patient care with medical research and education. This diversity in hospital types was considered in the sampling strategy to ensure a range of organizational contexts.

### Participants

In this study, we contacted a total of 25 nurses for an interview from different hospital types, with each nurse having varying levels of experience using CDSSs. Participants were selected using a combination of purposive and convenience sampling. We opted for a larger sample size because, while saturation in qualitative studies typically occurs with 6-12 interviews, more complex contexts often require 15-30 interviews to ensure comprehensive coverage of the subject matter [20]. In total, 22 nurses accepted the invitation for an interview. Saturation was monitored during the data collection process and was considered reached after approximately 18 interviews. The remaining interviews confirmed that no new themes were emerging across hospital types and professional roles. The remaining interviews added nuance or illustrative quotations only, and the codebook remained stable after the 18th interview, supporting the judgment that thematic saturation had been achieved. This diversity in backgrounds, roles, and hospital types allowed for a broad range of perspectives to be captured. The participants included practicing nurses, nurse-in-training, and clinical nurse information officers (CNIOs). Their experience with CDSSs varied considerably: some used specific CDSS tools regularly (eg, risk scoring), while others only knew the term CDSS or were exposed indirectly, such as through implementation projects or digital protocols. By incorporating nurses with experience ranging from 1 year to over 3 decades, we ensured the data reflects both novice and seasoned professionals’ experiences with CDSS usage. This variation in expertise, hospital types (general, top-clinical, and academic), and professional roles provides a rich and comprehensive understanding of the facilitators and barriers associated with CDSS use.

### Data Collection

A semistructured interview guide was developed based on the key constructs of the extended FITT framework: individual, task, technology, and organizational context. The selection of interview questions was informed by prior studies using the (extended) FITT framework in CDSS research [10,12-14], as well as broader models of technology adoption in health care, such as the UTAUT model (Table 1). This theoretical foundation ensured the alignment of our interview protocol with existing literature while allowing for an in-depth exploration of barriers and facilitators to CDSS use in hospital-based nursing practice. A semistructured approach was chosen to ensure flexibility, allowing participants to elaborate on their experiences while maintaining alignment with the research objectives. This method facilitated the identification of context-specific insights regarding CDSS implementation in nursing workflows.

**Table 1:** Themes and focus areas.

Theme	Focus area	Sample question
Individual	Nurses’ role, perception of CDSSs^a^, and attitudes toward technology	Which advantages or opportunities do you see in using clinical decision support tools in your daily practice?
Task	Decision-making processes and challenges with and without CDSSs	What disadvantages or barriers do you see in using clinical decision support tools for patient care?
Technology	System functionality, usability, and hospital support	How would you describe the support provided by the hospital for using digital systems in your work?
Organizational context	Leadership support and organizational culture around digital tools	What should the hospital address to improve (or further improve) support for the use of digital tools?

^a^CDSS: clinical decision support system.

Interviews were conducted in person alone at the participating hospital by one of three trained interviewers (Susan Bruggeling, Judit Putnoki, and Johanna Zuidhoff) with backgrounds in nursing and health informatics under the supervision of 2 researchers (MB and TVH). Their familiarity with clinical environments and training in health informatics helped build rapport with participants and ensure relevance of follow-up questions. At the same time, their background may have introduced implicit expectations about functionalities or the value of a digital system, potentially influencing how (follow-up) questions were asked or interpreted. This risk was mitigated through joint interview training and the use of a semistructured interview guide. All interviewers completed a training session together before the data collection commenced and conducted 2 pilot interviews each to refine the protocol, ensuring question clarity, consistency, and methodological rigor. These pilot interviews, which were discussed together, also allowed for the evaluation of question validity and comprehensibility from a nursing perspective. These interviews were excluded from the data analysis. A standardized interview protocol helped ensure consistency and minimize bias. The interviewers did not have a prior personal or extended professional relationship with the participants, except for limited professional contact in isolated cases.

Each interview lasted 30 to 60 minutes and was audio-recorded with participants’ written informed consent. The participant was interviewed a single time. Field notes from the interviewer were not gathered during the interview. Data collection occurred over a 4-month period between September 2022 and December 2022, ensuring representation from nurses across different shifts, departments, and experience levels.

### Data Analysis

The recorded interviews were transcribed verbatim and were then analyzed. The transcribed interview was sent back to the participant for a check, but no member check was conducted. Our analysis process is based on the 6 steps outlined by Braun and Clarke [21].

Steps 1 and 2, following Braun and Clarke’s approach, consisted of open coding conducted with an inductive first approach to identify factors directly emerging from the data. This initial step allowed us to ground the findings in participant experiences without being constrained by predefined frameworks, ensuring that all relevant elements from the interviews were captured.

Steps 3 and 4 from Braun and Clarke were simultaneously started in which the identified factors were directly after they were identified systematically plotted onto the FITT framework in seven categories using a deductive approach: (1) the fit and (2) no fit between “technology and individual,” (3) fit and (4) no fit between “task and technology,” (5) fit and (6) no fit between “individual and task,” and (7) “organizational context.” This 2-step simultaneous process ensured a balance between inductive coding for emerging factors and deductive coding rooted in the FITT framework. After we started with step 5, the identified factors were categorized as facilitators or barriers. This step involved analyzing how each factor influenced the adoption and implementation of CDSSs within the context of the FITT framework. In the last step (6), facilitators were defined as elements that promoted or enhanced CDSS use, while barriers were identified as factors that hindered the usage of CDSSs.

The analysis was conducted in Atlas.ti (v24; Lumivero) by 2 researchers (MB and SB) independently to reduce interpretive bias and to ensure the reliability of the findings. The researchers had a background in health care and informatics. Any discrepancies between the researchers were resolved after the coding through discussion by the 2 researchers until consensus was reached. After the analysis, an external expert session was conducted to validate the identified codes found by 2 independent researchers (MB and SB). The session lasted for 2 hours and involved 5 participants: 2 researchers (TVH and DS) with expertise in the health care domain, 1 researcher (KS) with experience in qualitative coding, 1 PhD student (MB), and 1 research assistant (SB). During the session, the group critically reviewed the codes, discussed their relevance, and ensured that they accurately reflected the data to find consensus. This collaborative approach allowed for a robust validation process, ensuring that the coding framework was aligned with the domain expertise of the participants [22].

Following the thematic analysis, we conducted a post hoc analysis to explore the value tensions inherent in the identified factors. Value tensions refer to conflicting priorities, needs, or expectations that emerge during the adoption and implementation of CDSSs, often reflecting deeper ethical, organizational, or professional dilemmas [23]. The value tensions were analyzed by re-examining the coded data through the lens of key ethical and practical trade-offs reported in prior literature on health informatics and digital health care adoption.

### Ethical Considerations

This research was conducted following Dutch human subject regulations. Since the Dutch Medical Research Involving Human Subjects Act (in Dutch “WMO”) did not apply, no official ethical approval was required. To confirm this assessment, the research team completed the decision tree for non-WMO research, as provided by the medical ethics review committee, which is aligned with national ethical guidelines [24]. The outcome confirmed that the study was classified as non-WMO. The act applies only to medical research that infringes on a subject’s physical or psychological integrity [25].

Although no official ethical approval was required, all necessary precautions were taken to protect participants’ anonymity and confidentiality. All participants were informed about the purpose and procedures of the study before their participation, including the nature of the questions to be asked, the intended use of the information they provided, and the fact that the data would be anonymized and kept confidential. The participants did not receive (financial) compensation for the interview. The data are stored according to a data management plan for this study and are not used in other studies. All participants had the opportunity to read back the transcript of the interview to check their data.

Written informed consent was obtained from all participants. They were assured that their participation was voluntary, and they had the right to withdraw from the study at any time without any repercussions. Care was taken to ensure that the interview setting was comfortable and nonintimidating, with interviews scheduled at times convenient for the participants, within their own hospital, to minimize disruption to their work schedules.

During the data analysis, all identifying information was removed to ensure participant anonymity. Access to the data was restricted to the research team, and all data were securely stored in accordance with the General Data Protection Regulation. The findings are reported in a way that individual participants could not be identified, ensuring their privacy and confidentiality throughout the research process.

## Results

### Participants’ Characteristics

A total of 22 nurses participated in the study. Participants were drawn from general, top-clinical, and academic hospitals across the Netherlands, ensuring variation in organizational settings. The sample included nurses with a range of clinical backgrounds and levels of experience, including practicing nurses, nurses-in-training (n=7), CNIOs, and medical technology officers. Participants’ experience with CDSSs varied, from those who used such systems daily to those involved in implementation or training efforts.

Participants were recruited from general, top-clinical, and academic hospitals, reflecting the range of hospital types in the Dutch health care system. Table 2 summarizes the characteristics of the participants.

**Table 2:** Characteristics of participants.

ID	Role	Hospital type	Years of experience
1	Nurse	General	13
2	Nurse or CNIO^a^	General	7
3	Nurse	General	22
4	Nurse and medical technology quality officer	General	14
5	Nurse	General	14
6	Nurse-in-training	General	14
7	Nurse	General	10
8	Nurse-in-training	General	N/A^b^
9	Nurse	Top-clinical	N/A
10	Nurse	Academic	32
11	Nurse or CNIO	Top-clinical	5
12	Nurse and practice trainer	Top-clinical	5
13	Nurse and practice trainer	Top-clinical	7
14	Nursing researcher	Top-clinical	7
15	Nurse	Top-clinical	1.5
16	Nurse or CNIO	Top-clinical	5
17	Nurse	Top-clinical	35
18	Nurse-in-training	Top-clinical	3
19	Nurse-in-training	General	3
20	Nurse	Top-clinical	36
21	Nurse-in-training	Top-clinical	4
22	Nurse	Top-clinical	38

^a^CNIO: chief nurse information officer.

^b^N/A: not available.

### The Fit of Clinical Decision Support Systems

Thematic analysis revealed 26 distinct factors that influence the adoption and use of CDSSs by nurses. These factors were categorized across the 4 FITT domains: *technology-individual fit*, *technology-task fit*, *task-individual fit*, and a redefined category of *organizational context*. During the coding process, 988 code instances were identified (Table 3). This number reflects the volume and depth of qualitative data analyzed across all interviews, including recurring themes and insights. However, our reporting focuses on the consolidated factors, as these represent the core themes relevant to understanding facilitators and barriers. Each factor was assigned based on thematic relevance rather than frequency, in line with qualitative best practices [21].

**Table 3:** Code frequency.

FITT^a^ group	Code frequency (n=988)
Task-individual	95
Technology-individual	407
Technology-task	433
Organizational context	53

^a^FITT: Fit Between Individuals, Tasks, and Technology.

We examined the identified factors to determine whether each factor serves as a facilitator (coded as “fit”), a barrier (coded as “no fit”), or both (coded as “fit and no fit”). Our analysis revealed that 10 factors function exclusively as facilitators, 7 factors operate solely as barriers, and 8 factors were coded as both facilitators and barriers. Table 4 presents the identified factors with an explanation.

**Table 4:** Identified factors.

Factor			Classification		Explanation
**Technology-individual fit**					
	Acceptance	Facilitator and barrier		Acceptance relates to the degree to which the CDSS^a^ is accepted, adopted, and used by nursing staff. This acceptance extends beyond technical usage and includes behavioral and cultural changes that occur during implementation. “Look, if you really look at what it can deliver, what it is, I think it has a lot of future” [ID7]. This quote underscores some nurses’ acceptance of the system. However, a lack of involvement leads to mistrust.	
	Autonomy	Facilitator and barrier		“Autonomy” addresses concerns about the system undermining nurses’ autonomy and competency. It includes worries about potential inability to deviate from the system and possible overreliance on the system. However, autonomy can also be enhanced as reliance on more experienced colleagues decreases. “But suppose you would have something like that, that makes multiple choices for you for a decision, that of course helps a lot. It also makes you independent in your work” [ID3].	
	Cognition	Facilitator		Cognition refers to the mental processes involved in acquiring knowledge and comprehension, including thinking, knowing, remembering, judging, and problem-solving. “Increase knowledge level. You don’t always have time to stay up to date, but in this way, you get the latest knowledge, I think” [ID8].	
	Clarification	Facilitator		Simplifies complex data, enabling nurses to focus on critical tasks by making information accessible and actionable. “You actually want to have everything at a glance” [ID5].	
	Digital proficiency	Barrier		Lack of adequate digital skills among nurses limits the system's usability and creates resistance to adoption. “...And often, these people, in healthcare in general, are there because they want to help people specifically, not because they enjoy working with computers” [ID7].	
	Support	Facilitator and barrier		Availability of resources enhances CDSS usage (*facilitator*), but insufficient support from the organization hinders its full potential (*barrier*). “...Who get eight hours a week to think about these kinds of things, including ICT. As a result, you naturally get much better representation of healthcare in ICT” [ID11].	
	Training	Facilitator and barrier		Adequate training programs facilitate CDSS adoption (*facilitator*), but gaps in training delivery leave some users unprepared (*barrier*). “Courses are offered, and there is careful attention given to training. However, as I mentioned before, I think more time needs to be allocated from both sides—from the ICT support side and from our side—to make it work optimally” [ID4].	
**Technology-task fit**					
	Alarm fatigue	Barrier		Overwhelming or irrelevant alerts desensitize nurses, leading to cognitive overload and risks to patient safety. “And now, dismissing an alarm is sometimes easier than accepting it, because accepting requires you to fill something in” [ID13].	
	Poor design	Barrier		Poorly designed systems fail to align with nursing workflows, creating inefficiencies and resistance. “The various devices cannot communicate with each other, but it still makes it somewhat possible” [ID2].	
	Usability	Facilitator and barrier		Intuitive and user-friendly designs improve workflow (*facilitator*), but poorly implemented usability features increase frustration (*barrier*). “You fill that in, and then it automatically prompts you to ask for the pain score again after an hour. However, it doesn’t yet take into account whether or not you’ve given pain medication” [ID9].	
	Information supply	Facilitator		Consolidates patient data into an easily accessible format, enabling timely and informed decision-making. “All the data combined in the system gives you an immediate and complete overview, allowing you to identify complications early on or facilitate timely handovers, for example” [ID3].	
	Continuous monitoring	Facilitator		Real-time tracking allows for early detection of patient deterioration, reducing response time and improving outcomes. “That there are also opportunities in having nurses who work partly in home care or visit patients in home situations, while also working on the ward. This way, you can follow the patient seamlessly across different locations” [ID14].	
	Reporting	Facilitator		The ability to generate reports increases visibility of patient outcomes and supports evidence-based practices. “So that you can work much more in a cycle where the actions of the nurses and their impact on an outcome become much more visible” [ID14].	
	Patient focused	Facilitator		Enables patient-centered care by supporting shared decision-making and improving patient outcomes. “Maybe that has to do with shared decision-making, right? So that a patient can also make better choices based on that support. Because indeed, I was reasoning very much from the perspective of the caregiver, but I can well imagine that the patient also gains more insight. For example, now they can already see for a surgery, You have this percentage chance of these complications, or similar information” [ID16].	
	Safety	Barrier		Concerns about overreliance on automation and potential system errors undermine trust and safety in critical situations. “Yes, for the patients and also my own safety. It cannot work 100% error-free, of course. That is indeed a downside or a risk” [ID6].	
	Restriction of freedom	Barrier		Overly rigid protocols restrict professional judgment and hinder the flexibility needed in unique cases. ”But this does ensure that you can, for example, not only insert a catheter but must also apply other types of interventions and therapies before actually inserting a catheter” [ID3].	
	Health care quality	Facilitator		“Health care quality” pertains to the degree to which health services increase the likelihood of desired health outcomes and are consistent with current professional knowledge. An illustrative quote is: “That you are supported with a lot of data for a better care experience, or not experience, but result. And that it signals that, it facilitates the work, accelerates the provision of care and also more guarantee for the patient that it goes well” [ID8].	
**Technology-individual fit**					
	Accountability	Facilitator		Facilitates transparent documentation, enabling nurses to justify their decisions with confidence. “Then I can simply use that as justification, right? Like, ‘This is a study they conducted, and it is documented here with this reasoning, and it states this...’ ” [ID2].	
	Workload	Facilitator and barrier		Reduces administrative burdens for some tasks (*facilitator*), but introduces additional work such as repetitive data entry (*barrier*). “The care process can be faster and more efficient, I think, because you immediately have all the information” [ID6].	
	Prevention	Facilitator		Enables early identification of potential risks, allowing nurses to intervene proactively. “You can act more quickly based on vital signs or previous medical conditions, guided by the advice and support provided by the system” [ID3].	
	Uniformity	Facilitator		Standardizes care delivery, reducing variability and ensuring consistent outcomes across nurses. “And everyone operates in the same way” [ID2].	
	Work ethic	Barrier		Risks of overreliance or loss of meaningful work elements negatively affect nurses' engagement and job satisfaction. “But yes, as I said, just checking off lists doesn’t automatically mean that you’ve provided good patient care” [ID9].	
	Contact	Facilitator and barrier		Frees up time to focus on patients (*facilitator*), but excessive automation may reduce personal interactions with patients (*barrier*). “Yes, it remains human work, and if you rely heavily on automation systems, it can sometimes become very impersonal, and you might end up missing things” [ID10].	
**Organizational context**					
	Change management	Facilitator and barrier		Properly managed transitions facilitate system adoption (*facilitator*), but poor change management creates resistance and confusion (*barrier*). “But then you see that it’s quite a long process to actually implement it because the tools are already there. However, the question becomes, who is going to do it? And then there’s the entire process of figuring out how to organize everything logistically” [ID10].	
	Management support	Facilitator		Strong leadership ensures smoother implementation, adequate training, and alignment of goals. “New things, when they want to try new things, I’m always curious about that, like, how does it turn out? It depends a bit on how they introduce it. If it’s just, ‘Get used to it, we’ve already decided, this is it, and we’re not changing anything even if it’s terrible,’ then...yeah. It starts off on the wrong foot” [ID18].	
	Cash	Barrier		Limited budgets prevent investment in training, support, and system upgrades, hindering adoption and usage. “Yes, we have a limited budget; we can’t just implement one of these tools without careful consideration” [ID7].	

^a^CDSS: clinical decision support system.

### Cross Synthesis

Taken together, patterns were role- and context-sensitive. Nurses-in-training described CDSSs as a scaffolding for decision-making and knowledge updating; experienced nurses stressed alert noise, extra clicks, and loss of flexibility; and CNIOs framed success around structured training and content governance. By hospital type, general hospitals more often emphasized basic usability and resource limits, while top-clinical hospitals highlighted interoperability, device or EHR integration, and alarm management. These differences help explain why the same functions could operate as facilitators in one setting and barriers in another.

### Value Tensions Across FITT Categories

In the final stage of our analysis, we examined how the identified factors shown in 4 interacted across the FITT domains and gave rise to underlying value tension situations where competing priorities or goals created friction. These tensions were not predefined but were identified inductively by examining patterns across coded excerpts that involved contrasting experiences or trade-offs described by participants and discussed by the researchers.

One key relationship is the interaction between autonomy (technology-individual fit) and restriction of freedom (technology-task fit). Participants described how CDSSs use could enhance autonomy by reducing their reliance on colleagues and supporting independent decision-making. However, they also noted that these same systems sometimes imposed constraints through rigid protocols or limited customizability, illustrating a tension between increased autonomy and loss of flexibility. For example, nurses expressed concerns about being unable to deviate from standardized recommendations when clinical judgment suggested an alternative course of action. This tension illustrates the need for CDSS designs that balance standardization with flexibility, allowing nurses to exercise their professional judgment while maintaining consistency in care delivery.

Another notable tension arises between uniformity (task-individual fit) and safety (technology-task fit). Uniformity, achieved through standardized processes and documentation, was viewed as a facilitator for reducing variability in care and ensuring consistent outcomes. However, overly rigid standardization may overlook unique patient circumstances, potentially compromising safety in complex or atypical cases. For example, nurses reported that while CDSSs provided valuable guidance for routine cases, their inability to account for nuanced patient conditions sometimes limited their applicability. Addressing this requires systems that allow for both adherence to best practices and customization to fit individual patient needs.

The relationship between management support (organizational context) and training (technology-individual fit) highlights the critical role of leadership in sustaining user engagement and system adoption. Strong leadership was identified as essential for providing adequate training resources and ensuring alignment between institutional goals and technology implementation. However, the absence of visible support from management often led to gaps in training, leaving nurses feeling unprepared to use CDSSs effectively. This finding emphasizes the interconnectedness of organizational and individual factors and the importance of a cohesive approach to system adoption.

The dual nature of workload (task-individual fit) also demonstrates how factors can function as both facilitators and barriers depending on context. CDSSs can reduce workload by streamlining routine tasks, such as documentation or early warning detection. Equally, it can introduce additional administrative burdens, such as repetitive data entry or managing excessive alerts. This tension highlights the need for workflow optimization, ensuring that CDSSs enhance efficiency without creating new inefficiencies.

A broader value tension lies in the interplay between patient-focused care (technology-task fit) and contact (task-individual fit). While CDSSs facilitate patient-centered care by supporting shared decision-making and improving health outcomes, participants expressed concerns that increased reliance on technology might reduce personal interactions with patients. Nurses reported that time spent navigating systems could detract from meaningful patient engagement, particularly in time at bed parts of care. Balancing these priorities requires designing systems that integrate seamlessly into workflows, minimizing the time spent on system interaction and maximizing direct patient care.

Lastly, the findings suggest that alarm fatigue (technology-task fit) interacts with cognition (technology-individual fit) in ways that affect user engagement. While CDSSs enhance cognition by simplifying data and providing actionable insights, excessive or irrelevant alerts can overwhelm nurses and reduce their ability to focus on critical information. This underscores the need for smarter alert algorithms that prioritize relevant notifications, ensuring nurses are directed toward meaningful tasks.

## Discussion

### Principal Results

This study examined the facilitators and barriers influencing the adoption and use of CDSSs within nursing practice in Dutch hospitals. The findings highlight 11 facilitators, 7 barriers, and 8 factors that are both facilitators and barriers. All factors were categorized according to the FITT framework. Beyond identifying these individual facilitators and barriers, the findings highlight value tensions, situations where competing priorities or perspectives create conflict within or between categories.

### Integration With Prior Research

The identified facilitators, barriers, and value tensions in CDSS adoption among nurses align with existing theories of health informatics adoption, such as the multidimensional nature of success described in Brender et al [26]. Similar to our use of the extended FITT framework, Brender et al emphasized that clinical and decision support systems are influenced not only by technical quality but also by organizational, behavioral, and management factors. In our study, the importance of factors like workflow alignment, user training, and leadership support shows their identified success dimensions: user involvement, management commitment, and fit with the organizational context. Moreover, several barriers we observed—such as alarm fatigue, lack of digital proficiency, and increased workload—reflect the types of failure criteria Brender et al highlight, including overloading the user, underestimating user acceptance, and not understanding the impact on work procedures. This reinforces earlier conclusions from our own rapid review on CDSS adoption in nursing, which identified similar facilitators and barriers, and emphasized the importance of aligning CDSSs with the values and realities of nursing practice [9]. Together, these findings demonstrate that while technology continues to advance, many foundational barriers to adoption remain persistent in practice.

Our findings also align with those of Meunier et al [8], who found that lack of integration with workflows, poor usability, and insufficient stakeholder engagement were persistent barriers in CDSS implementation, particularly in nursing and primary care settings.

Topaz et al [16] further reported that nurses often experience a misalignment between electronic health systems and their professional identity. Our findings present this, especially in relation to tensions between standardization and autonomy, or efficiency and meaningful patient contact. These relational and ethical aspects of CDSS use, rarely captured in earlier models, provide a novel perspective on adoption challenges.

However, our study also contributes new insights by connecting identified value tensions to the FITT framework. The identified tensions highlight the ethical and relational dimensions of CDSS adoption specific to nursing, suggesting that adoption is not only a technical or structural issue, but also a negotiation of values embedded in care practices.

This comparison underscores that while technology has evolved, many of the fundamental implementation challenges identified by earlier studies remain. Despite the passage of time, barriers related to user experience in IT systems, organizational change, and professional identity continue to shape CDSS adoption. This contribution lies in explicitly mapping these tensions across the FITT framework, offering a more dynamic view of the complexity of IT adoption. This reaffirms the need for more context-sensitive, participatory, and ethically adjusted approaches to implementation.

### Reflection on FITT Dimensions

The following section reflects on the main findings across each FITT dimension.

#### Technology-Individual Fit

The findings revealed that *acceptance* of CDSSs is highly contingent on trust and user involvement in the system’s design. While participants recognized the potential of CDSSs to streamline workflows and enhance decision-making, lack of involvement in system development emerged as a major barrier. This underscores the need for participatory design processes that engage nurses early in system creation. Additionally, *digital proficiency* remains a challenge for some, particularly for nurses with limited prior experience with technology, emphasizing the importance of targeted training programs. Experience-level asymmetries were clear: nurses-in-training emphasized cognitive scaffolding and confidence gains, whereas experienced nurses more often raised concerns about autonomy loss, supporting role sensitive design and training pathways rather than one-size-fits-all rollouts. *Cognition* and *clarification* were significant facilitators, as CDSSs simplified complex data and provided timely knowledge updates. These factors indicate that well-designed systems can enhance clinical confidence and reduce cognitive overload. However, inadequate *support* and inconsistent training hindered system adoption for many participants, suggesting that sustained organizational investment in user education and technical support is essential for long-term success.

#### Technology-Task Fit

While CDSSs demonstrated substantial potential to enhance *information supply*, *continuous monitoring*, and *reporting*, barriers such as *alarm fatigue* and poor *design* remained pervasive. Frequent, irrelevant alerts desensitize users and pose risks to patient safety, highlighting the need for advanced alert prioritization algorithms. Similarly, poorly aligned system interfaces exacerbated user frustration, emphasizing the importance of usability testing tailored to clinical workflows.

The system’s ability to support *patient-focused care* was identified as a key facilitator, enabling shared decision-making and improving patient outcomes. However, concerns around *restriction of freedom* and *safety* suggest that overly rigid protocols and potential system errors may undermine professional autonomy and trust. Developers must balance standardization with flexibility to address unique patient needs.

Alarm fatigue, as identified in this study, aligns with findings by Abell et al [27] which emphasize its impact on user desensitization. However, our research adds nuance by highlighting that nurses in critical care settings perceive this overload as exacerbated by the lack of integration between CDSS alerts and existing workflows. These findings reframe alert fatigue from a user-resilience issue to an organizational signal-engineering problem (thresholds, routing, escalation, and device or EHR integration), consistent with evidence that unmanaged alarms desensitize even experienced staff [28-30].

This underscores the importance of addressing workflow alignment alongside technical improvements.

#### Task-Individual Fit

Participants valued CDSSs for promoting *accountability* and *uniformity*, which reduced variability and enhanced care quality. However, the dual impact of *workload* emerged as a central theme. While CDSSs streamlined some tasks, additional administrative burdens often negated these efficiency gains. Future implementations should aim to optimize workflows by minimizing redundant data entry and integrating CDSSs seamlessly into existing processes.

The system’s role in *prevention* was widely acknowledged, particularly in its ability to identify early warning signs. However, concerns about *work ethic* highlight the risk of overreliance on CDSSs, potentially diminishing professional engagement and critical thinking. These findings suggest a need to reinforce the complementary role of CDSSs rather than allowing them to dominate clinical decision-making.

The interaction between accountability and workload presents another tension in this category. While accountability is facilitated by CDSSs through standardized documentation, the added burden of maintaining these standards may increase workload. Accordingly, standardization aids accountability but can also constrain professional judgment in atypical cases; guardrails with explicit override (and rationale capture) may offer a better balance than hard locks [26]. This dual effect reflects the need for careful workflow design to balance efficiency with accountability.

#### Organizational Context

Organizational factors played a significant role in shaping CDSS adoption. Strong *management support* was a key facilitator, as engaged leadership provided the necessary resources and alignment with institutional goals. In addition, limited budgets also mentioned as *cash*, hindered system upgrades and training opportunities, excessively affecting resource-constrained settings. Effective *change management* emerged as both a facilitator and a barrier. While structured planning and clear communication facilitated smoother transitions, poor management of change processes created resistance and uncertainty among staff. This highlights the importance of robust implementation frameworks that address user concerns and foster collective ownership of CDSSs.

The findings align with Kujala et al [10], who emphasized the role of strong leadership in enabling successful CDSS implementation. However, our study highlights a unique aspect: the critical role of consistent communication in sustaining nurse engagement throughout the implementation process. This nuance underscores the need for transparent leadership at every stage of adoption.

Taken together, these recommendations move beyond confirmation by specifying where and why common remedies (eg, more training and more standardization) succeed or fail in everyday nursing work, and by redirecting levers from individual users to organizational signal- and workflow-engineering.

### Implications for Practice

Based on the findings, several recommendations emerge for the successful adoption and integration of CDSSs into nursing practice. The recommendations are ordered from “quick wins” to “structured investments.”

#### Quick Wins

The first quick win is to improve acceptance and usability; it is essential to involve nurses at all stages of CDSS development and implementation. Providing opportunities for nurses to share their insights ensures that the system aligns with clinical workflows and minimizes resistance stemming from lack of involvement [31].

Targeted training programs that are tailored to the diverse digital proficiency levels among nurses are also critical. These programs should emphasize the complementary role of CDSSs, reinforcing critical thinking and professional autonomy while addressing knowledge gaps, particularly for less experienced nurses. Continuous education can help ensure that nurses feel confident and competent in their interactions with CDSSs, for example, by integrating data-driven competencies into nursing studies as mentioned by the American Association of Colleges of Nursing [32].

The last quick win is that organizations must also ensure nurses have access to technical and operational support. Clear communication channels and structured feedback loops are essential to identify and resolve user challenges. Allocating dedicated training time for nurses to engage with CDSSs and related tools before implementation can possibly further enhance their effectiveness and ease of use. Leadership support is also critical for developing a culture of innovation. Leaders must prioritize CDSS adoption by demonstrating its value to clinical practice and allocating resources.

#### Medium-Term Investments

A medium-term investment is the preventive potential of CDSSs, which should be leveraged to drive adoption by integrating tools that alert nurses to early warning signs of patient deterioration. Highlighting this capability can underscore the system’s role in improving patient outcomes. It is essential to address ethical and professional concerns by reinforcing the role of CDSSs as a support tool rather than a replacement for clinical expertise. By emphasizing its ability to enhance decision-making while preserving the human aspects of care, organizations can mitigate fears of overreliance and loss of meaningful work, as mentioned in multiple studies [33,34]. A responsible implementation approach that considers these elements, for example, value-sensitive design [35], can be used to ensure that CDSSs contribute positively to nursing practice and patient care.

Another medium-term investment that can be mitigated is alarm fatigue, a barrier to CDSS adoption [28-30]. This can be mitigated through the incorporation of advanced alert management systems. Developers should implement smarter algorithms that prioritize clinically relevant alerts, distinguishing between critical and noncritical notifications. Such improvements would reduce cognitive overload and enhance patient safety. Similarly, usability issues must be addressed through usability testing with frontline nurses during the design phase. Intuitive systems that integrate seamlessly into existing workflows can reduce redundant administrative tasks and minimize the burden of data entry, ultimately enhancing efficiency.

CDSS developers should aim to balance standardization and flexibility in system design. While CDSSs can enhance uniformity in care delivery, they must also accommodate unique clinical situations by allowing for overrides and customization. Supporting decision-making in this way ensures that nurses maintain professional autonomy and can reflect on past decisions to learn from previous cases.

#### Long-Term Structural Investments

While many actions are primarily enacted at the ward or team level, their impact ultimately depends on several system-level preconditions in Dutch hospitals. Hospitals should ensure that CDSS content and handovers align with nationally endorsed information standards for electronic exchange; plan integrations on a standards-based roadmap so device-EHR-CDSS interfaces are reliable and monitored; formalize hospital-wide governance for alerts and decision support (clear ownership of default settings, change control, and routine review of alert volumes, overrides, and time-to-action); and schedule go-lives with protected training time and backfill, acknowledging ongoing nurse-staffing pressures.

### Limitations

This study provides insights into the adoption and use of CDSSs by nurses in hospital settings; however, several limitations should be acknowledged.

First, the study focused exclusively on nurses’ perspectives, offering a detailed exploration of their experiences and challenges with CDSSs. While this single-disciplinary focus was essential to understand the specific interactions between nurses and CDSSs, it does not fully capture the interdisciplinary nature of CDSS usage in hospitals. Input from other stakeholders, such as physicians, administrators, and patients, could provide a more holistic understanding of the factors influencing adoption and implementation.

Second, while the extended FITT framework incorporated organizational context, this study did not explore specific organizational factors such as workflow dynamics, hierarchical structures, or institutional policies that may shape CDSS adoption. These organizational elements likely influence facilitators and barriers in significant ways, and future studies should consider a more granular analysis of these factors, focusing specifically on the organizational context.

Third, the qualitative design of this study enabled in-depth insights into nurses' lived experiences but limits the generalizability of the findings. Although the sample included nurses with diverse roles and experience levels from different hospital types, the findings remain context specific. Quantitative or mixed methods approaches could validate these findings and provide broader generalization. Furthermore, while this study was conducted within the Dutch health care system, many of the identified facilitators and barriers, such as alarm fatigue, training, usability, and leadership support, are relevant across other health systems. Nonetheless, differences in health care funding models, workforce structures, and digital infrastructure may influence the generalizability of our findings. In addition, while the data collection was over 2 years ago, insights remain applicable to ongoing implementation efforts as the core experiences and challenges described by nurses remain highly relevant.

Last, a principal limitation concerns our overlap of inductive coding and deductive FITT mapping, as during the analysis of the interviews, the factors and the fit or no fit aspects were coded simultaneously, combining the first two phases. Although this provided efficiency, we acknowledge that conducting open coding and deductive categorization in parallel may have introduced some risk of bias or reduced the emergence of unexpected themes. To address this, we initially applied an open inductive approach first to allow themes to surface directly from the data before aligning them with the FITT framework. This approach was further strengthened by an expert validation session to ensure that no relevant themes were constrained or overlooked. We acknowledge that subtle or emergent themes may still be underrepresented because of this approach. Nevertheless, we recommend that future studies consider separating inductive and deductive phases more clearly to enhance methodological rigor and reduce interpretive bias.

### Future Research Directions

Future research could expand this study by incorporating quantitative data collection to allow for broader generalization of the findings. Quantitative data could provide more precise insights into the extent and impact of various facilitators and barriers. Additionally, further research could explore variations in barriers across different settings or user groups, which may inform more tailored implementation strategies that address specific needs or challenges in CDSS usage.

Another promising future research direction is the application of decision mining as a quality assessment instrument. By retrospectively analyzing decisions made by nurses when using CDSSs, decision mining can offer valuable insights into decision-making patterns, the quality of clinical judgments, and potential areas for improvement. This retrospective approach not only provides a method to assess the effectiveness of CDSSs but also identifies where barriers may have impacted decision quality. These insights can be used to refine both the CDSS itself and the support systems around its implementation, ensuring that barriers, such as design or usability issues, are addressed. In doing so, decision mining could play a key role in optimizing CDSSs for nursing practice, improving both their adoption and effectiveness in enhancing patient care.

### Conclusions

This study highlights the factors influencing the adoption and use of CDSSs by nurses in hospitals, emphasizing the importance of achieving a fit between individuals, tasks, technology, and organizational contexts. The extended FITT framework provided a valuable lens for identifying facilitators and barriers that can guide practical improvements. By addressing critical areas such as user engagement, training, system usability, and organizational support, CDSS implementation can be more effectively tailored to meet the needs of nurses. These findings contribute to a growing understanding of how to optimize CDSS integration into nursing workflows, ensuring it enhances clinical decision-making and improves patient outcomes.

## Abbreviations

CDSS: clinical decision support system
CNIO: chief nurse information officer
EHR: electronic health record
FITT: Fit Between Individuals, Tasks, and Technology
IT: information technology
UTAUT: Unified Theory of Acceptance and Use of Technology
WMO: The Dutch Medical Research Involving Human Subjects Act

## Data Availability

The datasets generated or analyzed during this study are not publicly available, as participants provided consent for their data to be used solely for the purposes of this scientific publication. Data may be made available from the corresponding author upon reasonable request.

## References

[1] Osheroff JA, Teich J, Levick D, Saldana L, Velasco F, Sittig D, Rogers K, Jenders R (2012). Improving Outcomes With Clinical Decision Support: An Implementer's Guide.

[2] Sutton RT, Pincock D, Baumgart DC, Sadowski DC, Fedorak RN, Kroeker KI (2020). An overview of clinical decision support systems: benefits, risks, and strategies for success. NPJ Digit Med.

[3] Kohn LT, Corrigan JM, Donaldson MS (2000). To Err Is Human: Building a Safer Health System.

[4] Chen Z, Liang N, Zhang H, Li H, Yang Y, Zong X, Chen Y, Wang Y, Shi N (2023). Harnessing the power of clinical decision support systems: challenges and opportunities. Open Heart.

[5] Roshanov PS, Fernandes N, Wilczynski JM, Hemens BJ, You JJ, Handler SM, Nieuwlaat R, Souza NM, Beyene J, Van Spall HG C, Garg AX, Haynes RB (2013). Features of effective computerised clinical decision support systems: meta-regression of 162 randomised trials. BMJ.

[6] Khairat S, Marc D, Crosby W, Al Sanousi A (2018). Reasons for physicians not adopting clinical decision support systems: critical analysis. JMIR Med Inform.

[7] Ruland CM, Bakken S (2002). Developing, implementing, and evaluating decision support systems for shared decision making in patient care: a conceptual model and case illustration. J Biomed Inform.

[8] Meunier P, Raynaud C, Guimaraes E, Gueyffier F, Letrilliart L (2023). Barriers and facilitators to the use of clinical decision support systems in primary care: a mixed-methods systematic review. Ann Fam Med.

[9] Berkhout M, van Barneveld A, Smit K, van Houwelingen T, Pucihar A, Kljajić Borštnar M, Blatnik S, Marolt M, Bons RWH, Smit K, Glowatz M (2025). “Rage against the machine?”: the impact of clinical decision support systems on hospital nursing decision-making, workflow efficiency, and patient outcomes: a rapid review. 38th Bled eConference—Empowering Transformation: Shaping Digital Futures for All.

[10] Kujala S, Ammenwerth E, Kolanen H, Ervast M (2020). Applying and extending the FITT framework to identify the challenges and opportunities of successful eHealth services for patient self-management: qualitative interview study. J Med Internet Res.

[11] Ammenwerth E, Iller C, Mahler C (2006). IT-adoption and the interaction of task, technology and individuals: a fit framework and a case study. BMC Med Inform Decis Mak.

[12] Zhai Y, Yu Z, Zhang Q, Zhang Y (2022). Barriers and facilitators to implementing a nursing clinical decision support system in a tertiary hospital setting: a qualitative study using the FITT framework. Int J Med Inform.

[13] Tsiknakis M, Kouroubali A (2009). Organizational factors affecting successful adoption of innovative eHealth services: a case study employing the FITT framework. Int J Med Inform.

[14] Noblin A, Shettian M, Cortelyou-Ward K, Schack Dugre J (2017). Exploring physical therapists' perceptions of mobile application usage utilizing the FITT framework. Inform Health Soc Care.

[15] Ye J, Woods D, Bannon J, Bilaver L, Kricke G, McHugh M, Kho A, Walunas T (2022). Identifying contextual factors and strategies for practice facilitation in primary care quality improvement using an informatics-driven model: framework development and mixed methods case study. JMIR Hum Factors.

[16] Topaz M, Ronquillo C, Peltonen L, Pruinelli L, Sarmiento RF, Badger MK, Ali S, Lewis A, Georgsson M, Jeon E, Tayaben JL, Kuo C, Islam T, Sommer J, Jung H, Eler GJ, Alhuwail D, Lee Y (2016). Nurse informaticians report low satisfaction and multi-level concerns with electronic health records: results from an international survey. AMIA Annu Symp Proc.

[17] Newton N, Bamgboje-Ayodele A, Forsyth R, Tariq A, Baysari MT (2025). A systematic review of clinicians' acceptance and use of clinical decision support systems over time. NPJ Digit Med.

[18] Fernando M, Abell B, Tyack Z, Donovan T, McPhail SM, Naicker S (2023). Using theories, models, and frameworks to inform implementation cycles of computerized clinical decision support systems in tertiary health care settings: scoping review. J Med Internet Res.

[19] Hill A, Morrissey D, Marsh W (2024). What characteristics of clinical decision support system implementations lead to adoption for regular use? A scoping review. BMJ Health Care Inform.

[20] Guest G, Bunce A, Johnson L (2006). How many interviews are enough?. Field Methods.

[21] Braun V, Clarke V (2008). Using thematic analysis in psychology. Qual Res Psychol.

[22] Naeem M, Ozuem W, Howell K, Ranfagni S (2023). A step-by-step process of thematic analysis to develop a conceptual model in qualitative research. Int J Qual Methods.

[23] Goodman KW (2020). Ethics in health informatics. Yearb Med Inform.

[24] Is review required?. NedMec.

[25] Central Committee on Research Involving Human Subjects.

[26] Brender J, Ammenwerth E, Nykänen P, Talmon J (2018). Factors influencing success and failure of health informatics systems. Methods Inf Med.

[27] Abell B, Naicker S, Rodwell D, Donovan T, Tariq A, Baysari M, Blythe R, Parsons R, McPhail SM (2023). Identifying barriers and facilitators to successful implementation of computerized clinical decision support systems in hospitals: a NASSS framework-informed scoping review. Implement Sci.

[28] Lewandowska K, Weisbrot M, Cieloszyk A, Mędrzycka-Dąbrowska W, Krupa S, Ozga D (2020). Impact of alarm fatigue on the work of nurses in an intensive care environment—a systematic review. Int J Environ Res Public Health.

[29] Chromik J, Klopfenstein SAI, Pfitzner B, Sinno Z, Arnrich B, Balzer F, Poncette A (2022). Computational approaches to alleviate alarm fatigue in intensive care medicine: a systematic literature review. Front Digit Health.

[30] Bell L (2010). Monitor alarm fatigue. Am J Crit Care.

[31] Paulsen MM, Varsi C, Andersen LF (2021). Process evaluation of the implementation of a decision support system to prevent and treat disease-related malnutrition in a hospital setting. BMC Health Serv Res.

[32] (2021). The essentials: core competencies for professional nursing education. American Association of Colleges of Nursing.

[33] Knop M, Mueller M, Kaiser S, Rester C (2024). The impact of digital technology use on nurses' professional identity and relations of power: a literature review. J Adv Nurs.

[34] Liberati EG, Ruggiero F, Galuppo L, Gorli M, González-Lorenzo M, Maraldi M, Ruggieri P, Polo Friz H, Scaratti G, Kwag KH, Vespignani R, Moja L (2017). What hinders the uptake of computerized decision support systems in hospitals? A qualitative study and framework for implementation. Implement Sci.

[35] Friedman B, Hendry D (2019). Value Sensitive Design: Shaping Technology With Moral Imagination.

